# Sp1 is involved in H_2_O_2_-induced PUMA gene expression and apoptosis in colorectal cancer cells

**DOI:** 10.1186/1756-9966-27-44

**Published:** 2008-09-24

**Authors:** Xinying Wang, Jing Wang, Shiyong Lin, Yan Geng, Jide Wang, Bo Jiang

**Affiliations:** 1 Department of Gastroenterology, Nanfang Hospital, Southern Medical University, Guangzhou, PR China

## Abstract

**Background:**

Reactive oxygen species (ROS) are intricately involved in tumor progression through effects on proliferation, apoptosis and metastasis. But how ROS works is not well understood. In previous study, we found PUMA (p53-upregulated modulator of apoptosis) played an important role in oxaliplatin-induced apoptosis. In the present study, we detect the role of PUMA in H_2_O_2_-induced apoptosis in colorectal cancer cells and investigate the potential mechanism.

**Methods and results:**

We showed that H_2_O_2 _stimulated the activity of a 493 PUMA promoter reporter gene construct. Suppressing the expression of PUMA abrogated H_2_O_2_-induced apoptosis. Deletion of the Sp1-binding sites also decreased the transactivation of PUMA promoter by H_2_O_2_. Furthermore, induction of PUMA promoter activity by H_2_O_2 _was abrogated by PFT-α (a p53 inhibitor) and Mithramycin A (a Sp1 inhibitor), as compared with PFT-α alone. To determine the effects of Sp1 on PUMA in H_2_O_2_-induced apoptosis, procaspase 3, procaspase 9 and procaspase 8 expression was assessed. Mithramycin A and PFT-α also reduced H_2_O_2_-induced apoptosis synergistically and abrogated the expression of procaspase 3 and procaspase 9.

**Conclusion:**

Our findings suggest that PUMA plays a role in H_2_O_2_-induced apoptosis, and that Sp1 works together with p53 in the regulation of H_2_O_2_-induced PUMA expression and apoptosis in colorectal cancer cells. This study provides important regulatory insights in the mechanisms of ROS in colorectal cancer.

## Introduction

Recently, a large body of evidence indicates that ROS plays a central role in intracellular and intercellular signal transduction pathway in a variety of cellular process. Reactive oxygen species (ROS) increased in colorectal cancer due to increased aerobic metabolism and exposure to various anti-cancer modalities such as ionizing radiation and chemotherapeutic drugs [1]. Many factors are involved in this process. ROS are capable of activating certain transcription factors directly and thereby modulating the regulation of gene transcription. Several transcriptional factors such as AP1, Sp1 [2,3], Smad [4] and snail are potentially associated with ROS-triggered cellular process. Apoptosis and cancer are opposed phenomena but ROS have been widely reported to play a key role in both[5,6], suggesting that the regulation of gene expression by oxidants, antioxidants and the redox state remains as a promising therapeutic approach. Hyperphysiological levels of ROS cause DNA damage, mutation and activation of several proto-oncogenes in normal cells [7,8]. On the other hand, the DNA damage and initiation of signal transduction pathways caused by ROS contribute to the cytotoxicity to cancer cells [9]. The mechanism involved is still controversial and its ability to induce apoptosis in colorectal cancer is not yet fully understood.

It is generally recognized that oxidative stress is associated with p53-dependent cell cycle arrest, DNA repair and apoptosis [10,11], but a clear understanding of the downstream regulation mechanisms is still elusive. It has been proposed that Bcl-2 regulates antioxidant pathways at sites of free radical generation [12]. Another gene, called p53 upregulated modulator of apoptosis (PUMA), was identified through global profiling as a p53-inducible gene. Yeast two-hybrid screening identified PUMA as a Bcl-2 interacting protein [13]. PUMA is a proapoptotic member of the Bcl-2 family and plays an important role in stress-induced apoptosis. Yu et al [13] suggested that PUMA could be directly activated by p53 through p53-responsive elements in its promoter region. The protein encoded by PUMA was exclusively localized to mitochondria where it interacted with Bcl-2 and Bcl-xl through its BH3 domain [14]. We have previously shown that oxaliplatin-induced ERK inactivation was involved in the regulation of oxaliplatin-induced PUMA expression and apoptosis [15]. We hypothesized that ROS had a direct effect on PUMA.

In the present study, we found that PUMA plays a role in H_2_O_2_-induced apoptosis in colorectal cancer cells. The effects of H_2_O_2 _on the expression of PUMA and the mechanism by which this is regulated were examined. Our results suggest that Sp1 plays a role in H_2_O_2_-induced PUMA expression and apoptosis in colorectal cancer cells. Comprehensive understanding of the ROS-triggered signal transduction, transcriptional activation and regulation of gene expression will help to identify the critical role of ROS in tumor progression and in defining a strategy for chemo-therapeutic intervention.

## Materials and methods

### Materials

All reagents for cell culture were purchased from Invitrogen/Life Technologies (Carlsbad, CA, USA). Mithramycin A (Mithr.A), pifithrin-alpha (PFT-α), Hoechest dye 33258, H_2_O_2 _and anti-PUMA antibody were purchased from Sigma-Aldrich (St-Louis, MI, USA). Anti-procaspase 3, 9, 8 antibodies were purchased from Cell Signaling (Beverly, MA). Anti-P53 (DO-1) antibody, anti-Sp1 (polyclonal antibody PEP2) antibody, anti-actin antibody and secondary antibodies were purchased from Santa Cruz Biotechnology (Santa Cruz, CA). Sp1 siRNA, control siRNA, siRNA Transfection reagent and siRNA Transfection Medium were purchased from Santa Cruz Biotechnology. All other chemicals were of analytical grade.

### Plasmids

The -336/+157 and -36/+157 PUMA-Luc reporter plasmids were a kind gift from Dr. Bert Vogelstein (Johns Hopkins University, Baltimore, MD, U.S.A.). The (-336/+157 -126/-25) PUMA-Luc plasmid was constructed by digesting the -336/+157 PUMA-Luc plasmid with Sac II and SmalI, and re-ligation according to reference 16. pSVβ-Galactosidase plasmid was purchased from promega.

### Cell culture, transient transfections and luciferase assays

The human colorectal cancer cell lines LoVo and HCT116 were obtained from American Type Culture Collection (Manassaas, VA), LoVo PUMA_AS cells was established as described in a previous study[15]. The LoVo cells and LoVo PUMA_AS cells were cultured in 25 cm^2 ^flasks in 1640 medium contained 10% (v/v) FBS with or without G418 (600 μg/ml). Cells were maintained at 37°C in a humidified 5% CO_2 _atmosphere until confluency and subcultured (1:10 split ratio) using trypsin (0.05% w/v)/EDTA (0.02% w/v). Cells for examination were grown in six-well cluster dishes. All treatments were carried out on cells at 70–80% confluence, PFT-α (20 μM) and Mithr.A (200 ng/ml) were added an hour before addition of H_2_O_2_.

Transient transfection was performed in 6-well plates using Lipofectamin 2000(Invitrogen) according to the manufacture's instructions. Cells were transfected with 0.5 μg luciferase reporter plasmid and 0.1 μg pSVβ, transfections were allowed to proceed for 3 hours in the absence of serum and the cells recovered in medium supplemented with reduced serum level(2.5%) for 21 hours. Luciferase assays were performed 48 hours later using the luciferase assay kit from Promega, according to the manufacture's instructions (Promega, Madison, WI). The β-galactosidase reporter pSVβ (Promega) was included to control for transfection efficiency.

### Reverse transcriptase-polymerase chain reaction

0.5 μg total RNA was isolated from the cells using TRIZOL (Gibco). PUMA mRNA expression was determined by RT-PCR analysis using a RevertAid First Strand cDNA Synthesis Kit (MBI) and PCR kit (SBS Genetech Co. Ltd., Beijing, China). The two gene-specific primers used for amplification were as follows: (upper) gacgacctcaacgcacagta and (lower) ccagggtgtcaggaggtg. PCR products were electrophoresed in 1.5% agarose gel. Beta-actin mRNA was also amplified as an internal control. The experiment was performed twice.

### Western blotting analysis of PUMA, p53, Sp1, procaspase 3,9 and procaspase 8 expression

Western blotting analysis was assessed according to the protocol described before[15]. In brief, cells were washed with ice-cold PBS twice and lysed with ice-cold lysis buffer. Protein samples (15 μg) were separated by SDS/PAGE (12% acrylamide gel) using a Bio-Rad Mini-Protean III system. Proteins were transferred to PVDF membranes and the membranes were blocked for 1 hour at room temperature with TBST. Blots were then incubated at room temperature with primary antibodies (1:500 dilution) and then primary antibodies were removed and the blots were extensively washed with TBS/Tween 20 for three times. Blots were then incubated for 1 hour at room temperature with the secondary antibodies (1: 2000 dilution). Then blots were extensively washed as above for 1 hour and developed using the Enhanced Chemiluminescence detection system and quantified using the GeneTools systems.

### siRNA transfection

siRNA transfection was performed according to the manufacture's instructions. In brief, LoVo cells were seeded at a density of 2 × 10^5 ^per well in 2 ml antibiotic-free 1640 medium supplemented with 10% FBS in a six well tissue plate. The cells were incubated at 37°C in a CO_2 _incubator until the cells were 70% confluent. siRNA duplex solution (6 μl of siRNA duplex in 100 μl siRNA Transfection Medium) was added directly to the dilute Transfection Reagent (6 μl of siRNA Transfection Reagent in 100 μl siRNA Transfection Medium). The mixture was mixed and incubated at room temperature for 30 minutes. Then the mixture and 0.8 ml siRNA Transfection Medium were mixed and added to the cells. The cells were incubated 6 hours and the mixture was removed. H_2_O_2 _(0.64 mM) was added 24 hours later and remained for 15 minutes. Western blotting analysis was performed 48 hours post-transfection.

### Hoechst 33258 staining for apoptosis

For analysis of apoptosis by nuclear staining with Hoechst dye 33258 (Sigma Chemical Co.), cells were stimulated according to experimental protocols, washed once with PBS, and then fixed with pre-cooled methanol 500 μl/well for 10 minutes. After fixation, cells were washed once with PBS, stained with 1 μM Hoechst dye 33258 for 10 minutes, and then washed once with PBS and distilled water. Apoptosis was indicated by the presence of condensed or fragmented nuclei which bind the Hoechst dye 33258 with high affinity. For analysis of cell structure by photomicroscopy, coverslips were washed once with PBS and then inverted and mounted on to glass slides. Cells were visualized using an Olympus microscope. Two hundred cells in three randomly chosen fields were counted and scored for the incidence of apoptotic chromatin changes under fluorescence microscopy.

### Statistical analysis

All data are presented as mean values ± standard deviation (SD) of three independent experiments. Comparison of the effects of various treatments was performed using factor analysis, one-way ANOVA analysis of variance and a two-tailed *t*-test. Difference with a p value of < 0.05 were considered statistically significant.

## Results

### H_2_O_2 _induces time- and dose-dependent PUMA expression and apoptosis in colorectal cancer cells

**Figure 1:**
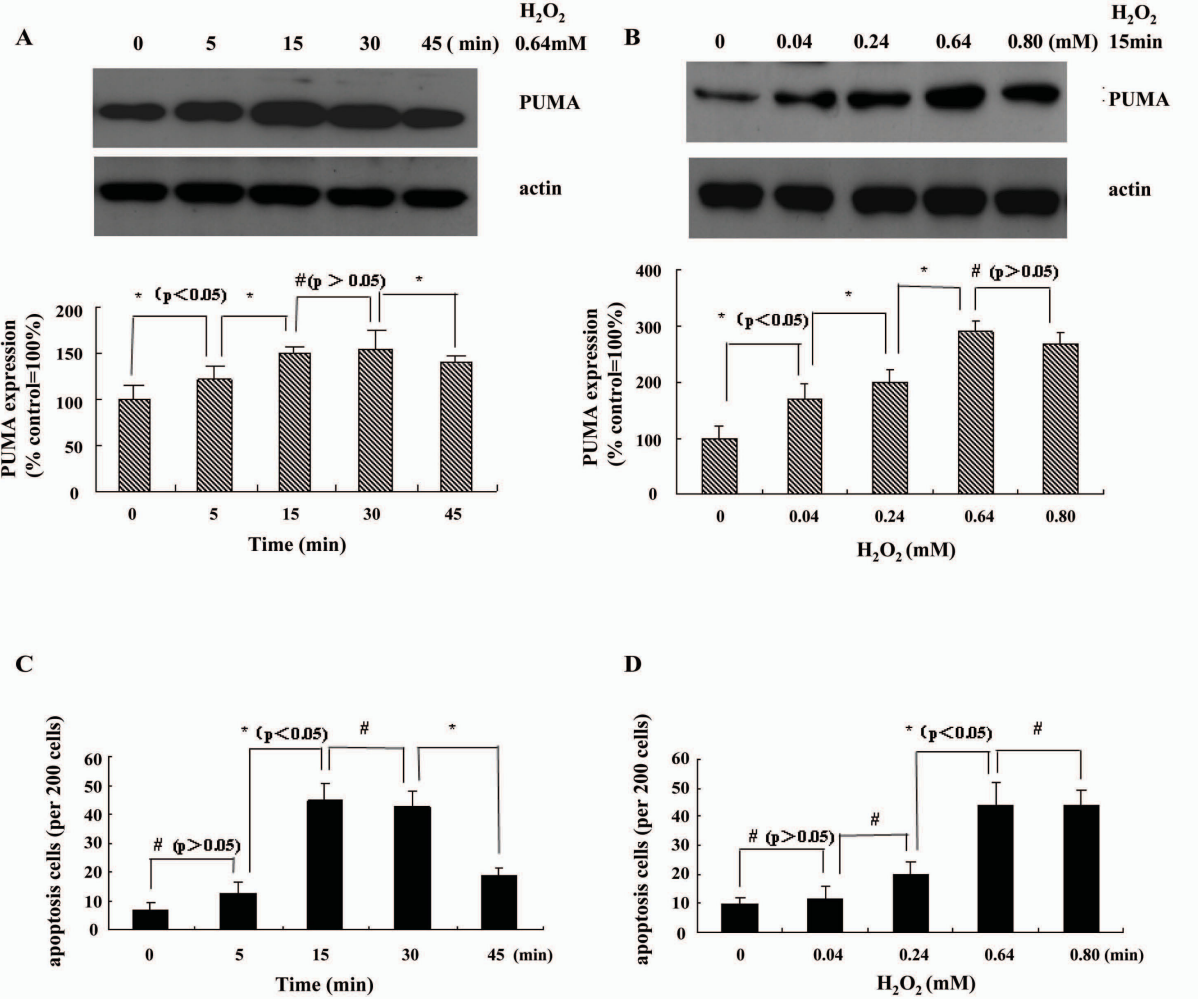
**H_2_O_2 _induced PUMA expression and apoptosis in a time-and dose-dependent manner in colorectal cancer cells.** (A) LoVo Cells were treated with 0.64 mM H_2_O_2 _at different time point and PUMA expression was assessed by Western blotting analysis. (B) Western blotting analysis of a dose-range of a 15 minute H_2_O_2 _exposure of LoVo cells. Data was presented as the percentage of the basal level of PUMA expression (control = 100%) in the absence of H_2_O_2 _and expressed as mean ± SD of three independent experiments. (C, D) LoVo Cells were treated with a time- and dose- range of H_2_O_2 _as indicated in Figure 1C, D, apoptosis was assessed by Hoechst dye 33258. Data was presented as the number of apoptosis per 200 cells in three different areas. Statistical significance as compared to control cells (*p < 0.05, ^#^p > 0.05).

To assess the effects of H_2_O_2 _on PUMA expression, we exposed LoVo cells to a dose range of H_2_O_2 _at different time points, and we measured PUMA expression by Western blotting analysis 24 hours later. Exposure to H_2_O_2 _was found to induce PUMA expression significantly already after 5 minutes. A maximal upregualtion by approximately 50% was observed after 15 minutes (Figure 1A). A dose-range identified that H_2_O_2 _induced PUMA expression maximally at a concentration of 0.64 mM (Figure 1B).

To assess the effects of H_2_O_2 _on apoptosis in colorectal cancer, we exposed LoVo cells to a dose range of H_2_O_2 _at different time points, and we measured apoptosis by Hoechst 33258 dye 24 hours later. As shown in Figure 1C, H_2_O_2_-induced apoptosis increased significantly at 15 minutes and decreased after 30 minutes. And apoptosis increased significantly when LoVo cells were treated for 15 minutes with 0.64 mM and 0.80 mM of H_2_O_2_. (Figure 1D).

### H_2_O_2 _up-regulates PUMA expression at the transcriptional level in colorectal cancer cells

**Figure 2:**
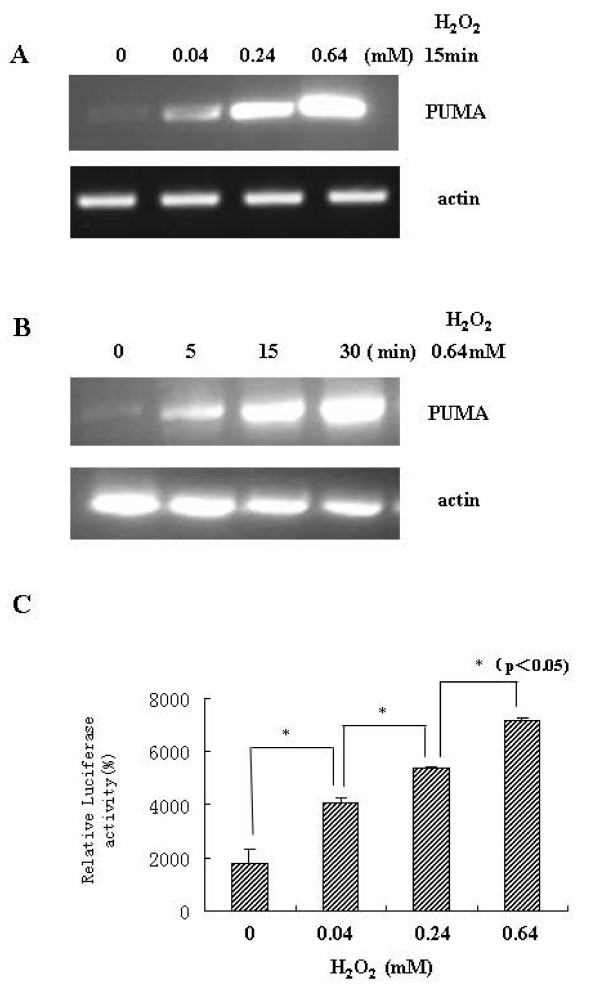
**H_2_O_2 _regulated PUMA expression at transcriptional level.** (A, B) Dose and time response of LoVo cells treated with H_2_O_2_. PUMA mRNA expression was detected by RT-PCR using 0.5 μg total RNA. Beta-actin mRNA was amplified as an internal control. (C) LoVo cells were transiently transfected with the -336/+157 PUMA-luc reporter plasmid (0.5 μg). H_2_O_2 _(0.64 mM) was added to the indicated samples 24 hours post-transfection and exposure was continued for 15 minutes. The pSVβ-Galactosidase plasmid (0.1 μg) was included in each sample for normalization of transfection variability. Luciferase activity was determined in cell lysates 48 hours following the transfection and the values (mean ± SD, *p < 0.05) from at least two independent expression performed in duplicate are shown in the form of a bargraph.

To investigate whether H_2_O_2 _increases PUMA expression at the mRNA level, we treated LoVo cells with a dose and time range of H_2_O_2 _and performed a standard RT-PCR experiment. As seen in Figure 2A and 2B, H_2_O_2 _increased PUMA mRNA in a dose- and time- dependent manner. Therefore, the data suggested that H_2_O_2 _up-regulated PUMA expression at transcriptional level.

Activation of p53 by e.g. DNA-damaging agents is followed by an induction of PUMA expression, leading to enhanced recruitment of endogenous p53 protein to the PUMA gene promoter. To test this, we used a -336/+157 PUMA-Luc reporter plasmid, which contains a set of 10 potential binding sites for Sp family proteins in the proximal region between nucleotides-120 and 18, flanked by two p53-recognition sequences at the 5' end between nucleotides -229/-209 and -145/-126. LoVo cells were transfected with this vector, 24 hours before H_2_O_2 _(0.64 mM) was added. As shown in Figure 2C, a significant transactivation of this promoter was observed after exposure to H_2_O_2 _for 15 minutes. These results suggest that H_2_O_2 _can influence endogenous PUMA levels by regulating PUMA promoter activity.

### H_2_O_2_-induced apoptosis is abrogated by suppression of PUMA expression

**Figure 3:**
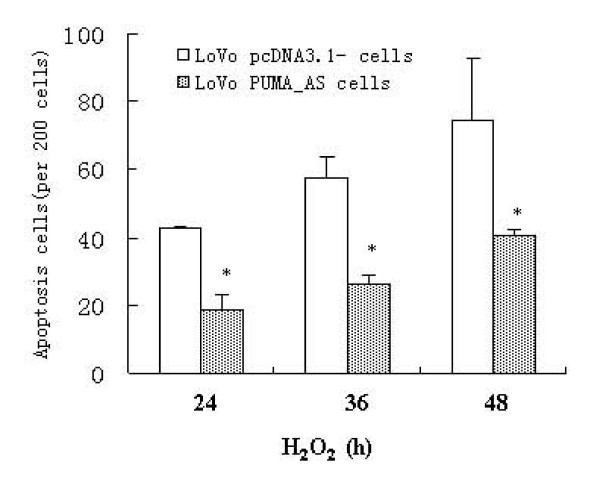
**Suppression of PUMA expression abrogated the apoptosis induced by H_2_O_2_.** LoVo PUMA wild-type cells and LoVo PUMA_AS cells were treated with H_2_O_2 _(0.64 mM) for 15 minutes and allowed to recover for 24 hours. Hoechst dye 33258 was used to detect apoptosis. Data are presented as mean number of apoptotic cells (mean ± SD) from three independent experiments. Significant difference between LoVo PUMA wild-type cells and LoVo PUMA_AS cells is indicated by * (p < 0.05).

To investigate whether PUMA is required for cells to undergo apoptosis, we made use of LoVo PUMA_AS cells, are stable cell line with low expression of PUMA[15]. We reasoned that these cells may differentially respond to oxidative stress as compared with their normal counterparts (colorectal cancer LoVo cells). As shown in Figure 3, induction of apoptosis by H_2_O_2 _is significantly hampered in LoVo PUMA_AS cells, as compared with induction in control LoVo cells (p < 0.05).

### Sp1 is required for the transactivation of the human PUMA promoter by p53

**Figure 4:**
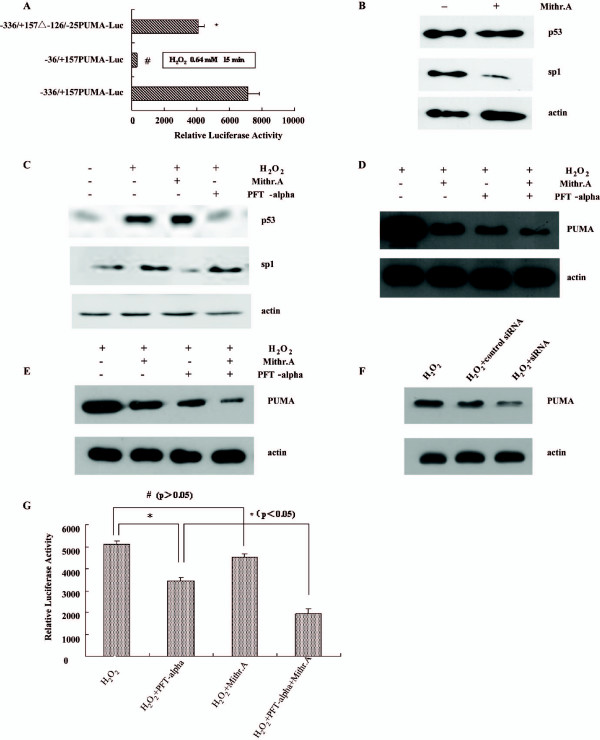
**Sp1 is required for the transactivation of the human PUMA promotor by H_2_O_2_.** (A) LoVo cells were transiently transfected with reporter plasmids (0.5 μg). 0.64 mM H_2_O_2 _was added 24 hours post-transfection for 15 minutes. The pSVβ-Galactosidase plasmid (0.1 μg) was included in each sample for normalization of transfection variability. Luciferase activity was determined in cell lysates 48 hours following the transfection and the values (mean ± SD), from at least two independent experiments performed in duplicate (*p > 0.05,-336/+157 -126/-25PUMA-Luc vs-136/+157 PUMA-Luc, ^#^p < 0.05,-36/+157PUMA-Luc vs-336/+157 -126/-25PUMA-Luc). (B) Mithr.A (200 ng/ml) was added to LoVo cells. Extracts from the treated or untreated LoVo cells were subjected to immunoblotting using antibodies against p53, Sp1 and actin. (C) LoVo cells were treated with 20 μM PFT-α and/or 200 ng/ml Mithr.A an hour before 0.64 mM H_2_O_2 _added. Cell extracts were analysed for the expression of endogenous p53, Sp1 and actin as indicated by immunoblotting. (D, E) LoVo and HCT 116 cells were treated with 20 μM PFT-α and/or 200 ng/ml Mithr.A an hour before 0.64 mM H_2_O_2 _added. Cell extracts were analysed for the expression of PUMA as indicated by immunoblotting. (F) H_2_O_2 _-induced PUMA expression was abrogated by transfecting Sp1 siRNA. LoVo cells were transfected with control siRNA or Sp1 siRNA and H_2_O_2 _was added 24 hours post-transfection. PUMA expression was detected by Western blotting analysis 48 hours post-transfection. (G) LoVo cells were transiently transfected with the -336/+157 PUMA-Luc reporter plasmid, 0.64 mM H_2_O_2 _was added 24 hours post-transfection and remained for 15 minutes. Mithr.A (200 ng/ml) and/or PFT-α (20 μM) was added an hour before H_2_O_2_. Luciferase activity was determined in cell lysates 48 hours following the transfection and the values (mean ± SD.) from at least two in dependent expression performed in duplicate are shown in the form of a bargraph, *p < 0.05, significant difference between cells treated as indicated in Figure 4G, ^# ^p > 0.05, no significant difference between cells treated as indicated in Figure 4G (factor analysis, n = 6).

The promoter of PUMA gene contains a cluster of GC-rich motifs flanked by two p53-recognition motifs at the 5' end. The p53 sites are located at distal regions relative to the proximal Sp1 sites. The family member Sp1 has been shown to mediate oxidative stress-induced gene transcription. To determine the role of Sp1 in H_2_O_2_-induced PUMA expression, we examined the activity of a series of PUMA promoter truncation mutants[16] after H_2_O_2 _treatment Compared with cells transfected with -336/+157 PUMA-Luc, the transactivation level of cells transfected with -336/+157 -126/-25PUMA-Luc was lower, but the difference was not significant (p > 0.05). Transfection with the -36/+157PUMA-Luc vector induced a significant decrease of transactivation (^#^p < 0.05, Figure 4A).

PFT-α is a stable, water soluble inhibitor of p53-dependent apoptosis and was also shown to reduce the activation of p53-regulated genes [17]. Mithramycin A (Mithr.A) is a drug which has been clinically used in the therapies of several type of cancer and Paget's disease. It binds to GC-rich regions in chromatin and interferes with the transcription of genes that bear GC-rich motifs in their promoters including Sp1[18]. Previous study suggested that Mithr.A also activates endogenous p53 protein and has no effect on the expression of endogenous Sp1 genes. To identify the effects of Mithr.A on p53 and Sp1 expression, Mithr.A (200 ng/ml) was added to LoVo cells 24 hours before p53 and Sp1 expression was assessed by Western blotting analysis. As shown in Figure 4B, Mithr.A had no effects on p53 expression, but suppressed the expression of Sp1. Mithr.A also abrogated the Sp1 expression induced by H_2_O_2 _(Figure 4C). Similarly, PFT-α (20 μM) abrogated p53 expression induced by H_2_O_2._

To determine the effects of Mithr.A and PFT-α on PUMA expression, Mithr.A and PFT-α were added an hour before H_2_O_2 _treatment. As shown in Figure 4D, PUMA expression was found to be suppressed by exposure to Mithr.A as well as to PFT-α, and more markedly when both compounds were combined. To determine whether these effects were cell-specific, we also assessed PUMA expression in HCT116 cells and gained the same results (Figure 4E). To confirm the role of Sp1 in H_2_O_2_-induced PUMA expression furthermore, we suppressed Sp1 expression by transfecting Sp1 siRNA and our data suggested that Sp1 siRNA abrogated H_2_O_2_-induced PUMA expression (Figure 4F).

To understand the mechanism of Mithr.A and PFT-α action, we tested the ability of Mithr.A and PFT-α to inhibit PUMA promoter activation induced by H_2_O_2_. As shown in Figure 4G, Mithr.A caused a decrease in PUMA promoter activity induced by H_2_O_2 _but the difference was not significant (p < 0.05). PFT-α, in contrast, caused a significant decrease in PUMA expression (p < 140.05). Combination of PFT-α and Mithr.A caused a significant enhancement of decrease in PUMA promoter activity (p < 0.05). Factor analysis (n = 6) indicated that Mithr.A and PFT-α have synergistic effects on H_2_O_2_-induced PUMA promoter activation (F = 5.480, p = 0.030).

### H_2_O_2_-induced apoptosis is abrogated by Mithr.A and PFT-α through a mitochondrial pathway

**Figure 5:**
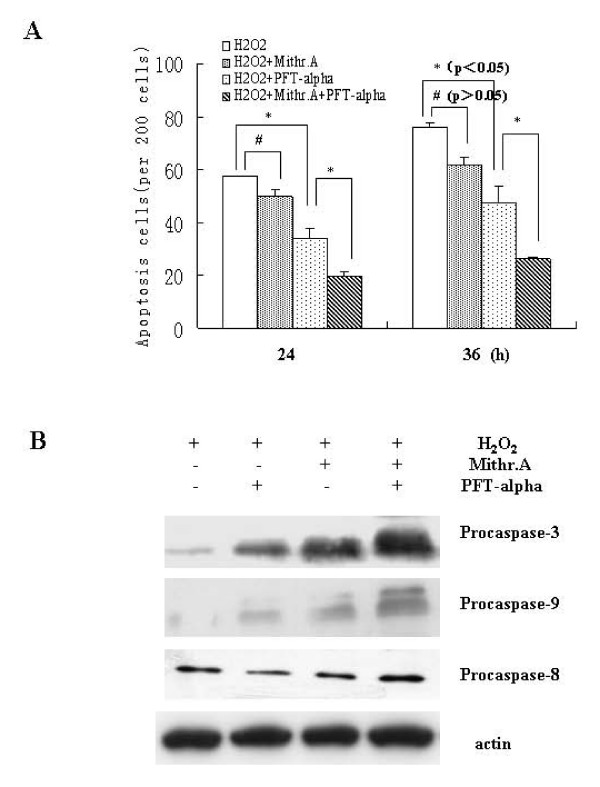
**PFT-α and Mithr.A decreased H_2_O_2_-induced apoptosis in a mitochondrial pathway.** (A) LoVo cells were treated with PFT-α (20 μM), Mithr.A (200 ng/ml) an hour before H_2_O_2 _(0.64 mM) was added. Hoechst dye 33258 was used to detect apoptosis (* represents significance p < 0.05, ^# ^represents significance p > 0.05). (B) LoVo cells were treated with PFT-α (20 μM), Mithr.A (200 ng/ml) an hour before H_2_O_2 _(0.64 mM) was added. Total extracts were then prepared and assayed for the level of the protein by immunoblot. Similar results were observed in two separate experiments.

To test whether a decrease in the expression of PUMA contributed to a decrease in the apoptosis of the cells, apoptosis assays were performed. We assessed the impact of Mithr.A and PFT-α treatment on H_2_O_2_-induced apoptosis in colorectal cancer cells. As shown in Figure 5A, Mithr.A caused a decrease in H_2_O_2_-induced apoptosis but the difference was not significant with untreated cells, while PFT-α caused a significant decrease. Compared with PFT-α alone, treatment with the combination of both compounds caused a significant decrease in H_2_O_2_-induced apoptosis.

PUMA is a mitochondrial protein involved in apoptosis in a mitochondrial pathway, leading to activation of caspase 9 and caspase 3 but not caspase 8. To assess the impact of Mithr.A and PFT-α treatment on these events, total cell extracts were prepared from H_2_O_2_, Mithr.A and PFT-α treated cells, and procaspase 3, procaspase 9 and procaspase 8 levels were monitored by Western blotting analysis. As shown in Figure 5B, increased levels of procaspase 3 and 9 were observed in Mithr.A and PFT-α treated cells, while effects on procaspase 8 were absent.

## Discussion

The generation of large amounts of reactive oxygen intermediates may contribute to the ability of some tumors to mutate, inhibit antiproteases and injure local tissues [19]. On the other hand, the production of ROS during the catabolism of tumor and chemotherapy leads to apoptosis and benefits in the process of the clearance of tumor cells. In the present study, we found that H_2_O_2 _up-regulated PUMA expression and induced apoptosis in colorectal cancer LoVo cells. Suppression of PUMA expression abrogated H_2_O_2_-induced apoptosis. To further elucidate the mechanism responsible for the changes in amounts of PUMA, we determined levels of PUMA mRNA by RT-PCR. Our data suggested that PUMA expression at the mRNA level increased and transcription activity of PUMA was upregulated by administration of H_2_O_2 _especially at doses between 0.04 and 0.64 mM in colorectal cancer LoVo cells.

PUMA is essential in the process of apoptosis induction by exogenous or endogenous p53 [20-22]. However, many studies described that there were other factors involved in the regulation of PUMA expression, either in a p53-dependent or independent manner including our previous report. For example, Tzippi et al [23] reported E2F1 upregulated the expression of PUMA through a direct transcriptional mechanism and increased PUMA levels mediated E2F1-induced apoptosis. Furthermore, Koutsodontis et al [16] found that the PUMA promoter contains a cluster of GC-rich motifs flanked by two p53-recognition motifs and a series of Sp1 recognition sites at the 5'-end. This was identified by searching the 5'-flanking sequence of PUMA with Tfsearch software . The same report also suggests that the Sp family was essential for the cellular response to p53 activation by genotoxic stress such as ROS [24,25]. This urged us to hypothesize that Sp1 plays a role in H_2_O_2_-induced PUMA expression and apoptosis.

To investigate the role of Sp1 on H_2_O_2_-induced PUMA expression, Mithr.A (a DNA-binding antitumor agent) [26] and PFT-α (a selective p53 inhibitor), were used for pharmacological intervention both in LoVo cells (p53 mutation) and HCT116 cells (p53 wide type). Sp1 siRNA was also used to confirm the result. Our data suggest that Sp1 plays a role in H_2_O_2_-induced PUMA expression and regulated it at the transcriptional level. This regulation is not cell specific in LoVo cells and HCT116 cells. To determine whether H_2_O_2 _could induce PUMA expression in p53 mutant cell lines, we detect the expression of PUMA in H_2_O_2_-treated SW480 cells(p53 mutated colorectal cancer cells) and LoVo p53- cells (established in our previous study). Our data showed that no much difference was found in H_2_O_2 _-treated cells and untreated cells (data not show) and this induction was p53-dependent.

Sp1 and p53 could regulate PUMA expression synergistically in H_2_O_2_-treated LoVo colorectal cancer cells. More interesting, Mithr.A was used as an antitumor agent and has been used in the clinic for several types of cancer. Lee et al [27] reported that it sensitized cancer cells to TRAIL-mediated apoptosis by down-regulation of XIAP gene promoter through Sp1 sites. Our data suggest that Mithr.A decreases apoptosis in colorectal cancer cells through inhibiting PUMA promoter activity, which may limit the application of Mithr.A in colorectal cancer therapy.

To assess whether inhibiting Sp1 binding to the PUMA promoter by Mithr.A contributed to a decrease of apoptosis induced by H_2_O_2_, Hoechst dye 33258 staining was carried out. Our data showed that combination of Mithr.A and PFT-α abrogated H_2_O_2_-induced apoptosis significantly better, as compared with PFT-α alone. PUMA induction results in Bax multimerizaion, mitochondia-dependent death, the release of cytochrome C and caspase 9 induction leading to apoptosis [14]. Our data showed an increase in expression of procaspase 3, procaspase 9 and suggested an decrease of caspase 3 and 9. Therefore, Sp1 activity may be functionally linked to the effects of H_2_O_2 _on PUMA.

In summary, a novel mechanism of PUMA stimulation by H_2_O_2 _in colorectal cancer LoVo cells has been demonstrated. The results suggest that H_2_O_2_-induced up-regulation of PUMA is partly due to Sp1 transcription factor except for p53. These results may provide an insight into the molecular control of H_2_O_2_-induced PUMA expression in colorectal cancer cells through Sp1 binding sites.

## Competing interests

The authors declare that they have no competing interests.

## Authors' contributions

WXY carried out Western blotting analysis, Hoechst 33258 staining for apoptosis, Statistical analysis and drafted the manuscript. WJ carried out the cell culture and Reverse transcriptase-polymerase chain reaction. LSY and GY carried out the transient transfections and luciferase assays. WJD constructed the plasmid. JB participated in its design and discussed the results. All authors read and approved the final manuscript.
